# Strain-Relief Patterns and Kagome Lattice in Self-Assembled C_60_ Thin Films Grown on Cd(0001)

**DOI:** 10.3390/ijms22136880

**Published:** 2021-06-26

**Authors:** Zilong Wang, Minlong Tao, Daxiao Yang, Zuo Li, Mingxia Shi, Kai Sun, Jiyong Yang, Junzhong Wang

**Affiliations:** School of Physical Science and Technology, Southwest University, Chongqing 400715, China; fragment@email.swu.edu.cn (Z.W.); taotaole@swu.edu.cn (M.T.); yangdx@email.swu.edu.cn (D.Y.); lizuo212@163.com (Z.L.); smx1215@email.swu.edu.cn (M.S.); skqtt@swu.edu.cn (K.S.); jyyang@swu.edu.cn (J.Y.)

**Keywords:** STM, C_60_, heptamer, kagome lattice, superstructure

## Abstract

We report an ultra-high vacuum low-temperature scanning tunneling microscopy (STM) study of the C_60_ monolayer grown on Cd(0001). Individual C_60_ molecules adsorbed on Cd(0001) may exhibit a bright or dim contrast in STM images. When deposited at low temperatures close to 100 K, C_60_ thin films present a curved structure to release strain due to dominant molecule–substrate interactions. Moreover, edge dislocation appears when two different wavy structures encounter each other, which has seldomly been observed in molecular self-assembly. When growth temperature rose, we found two forms of symmetric kagome lattice superstructures, 2 × 2 and 4 × 4, at room temperature (RT) and 310 K, respectively. The results provide new insight into the growth behavior of C_60_ films.

## 1. Introduction

The structures and growth process of C_60_ monolayer grown on metal surfaces have attracted widespread interest in the past decades due to the unique physical and chemical properties [1,2,3]. In the fullerene family, C_60_ was the first member to be created and most extensively discussed. C_60_ molecules form various self-assembled structures when deposited on different types of substrate, such as Au [4,5,6,7,8,9], Ag [10,11,12,13,14], Cu [15,16], Pb [17], Pd [18], P [19], Al [20], graphene [21,22,23,24], WSe_2_ [25], Si [26,27,28,29], and Ge [30,31,32]. Many results indicate that C_60_ molecules are easy to nucleate at the terrace edge and may form a close-packed structure under appropriate conditions, regardless of the types of substrate, such as the 2√3 × 2√3 R30° domain on Au(111) [33,34] and the 4 × 4 superstructure on graphene/Cu(111) [22].

Strain plays a central role in governing the structures of self-assembled thin films when organic molecules are deposited onto solid surfaces [35,36,37,38]. Strain derives from the competition between molecule-molecule interactions and molecule-substrate interactions. Currently, several strain relaxation mechanisms are known, such as step bunching [39], faceting [40], misfit dislocations [41,42], and the formation of periodic domain boundaries [43,44], due to the dominant molecule–substrate interactions of the different layers [45]. In the STM images of the C_60_ monolayer, the individual C_60_ molecules appear as a bright or dim contrast [4,5,6,7,8,9,10,11,12,13,14,15,16]. The various arrangements of the bright and dim C_60_ molecules combined with the multiple molecular orientations lead to the formation of a series of superstructures in the C_60_ monolayer, such as 2 × 2 [8,25,26], 3 × 3 [5,21], √19 × √19 [28,29], 7 × 7 [4,5,9], and a triple-stripe phase [18]. Moreover, the kagome lattices built from intersecting triangles and hexagons are observed in some C_60_ monolayers [8,13,23]. It is necessary to study the behavior of bright or dim contrast in kagome lattice when the C_60_ thin film is under strain.

In divalent metal Cd, there is a Coulomb repulsion between the 4d electrons and conduction band electrons, which leads to electrons with strong anisotropy in the mean free path and heat conduction. Recently, the Cd(0001) surface was shown to be a good substrate for observing and investigating strain phenomena of epitaxial films [46,47].

In this study, we used Cd(0001) thin films grown on Si(111)-7 × 7 as a substrate to explore the interface structures of the C_60_-Cd system. The STM study demonstrated that the C_60_ thin films on the Cd(0001) surface presented diversity at different growth temperatures. When the growth temperature was close to 100 K, an unexpected wavy structure driven by compressive strain appeared. Two lines of the molecular arrangements were curved and corresponded to the √3 directions of the Cd(0001) surface. Moreover, an edge dislocation was observed when two neighboring wavy domains with different directions were encountered. When the growth temperature increased, two kinds of symmetric kagome lattice, superstructures (2 × 2 and 4 × 4) were identified in the C_60_ domains, where individual C_60_ molecules exhibited a bright or dim contrast.

## 2. Results and Discussion

### 2.1. An Individual C_60_ Seven-Molecule Cluster at Two Bias Voltage

**Figure 1:**
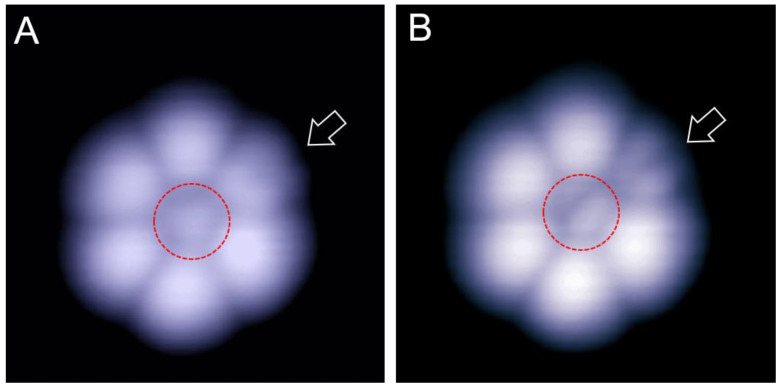
Isolated C_60_ heptamer form on the Cd(0001) surface at 100 K. (**A**,**B**) The empty-state STM images (6 × 6 nm) recorded at 1.2 and 0.5 V, respectively. The central molecule which is marked by a red circle manifests a triangle shape at 1.2 V(**A**), and a two-lobe motif at 0.5 V (**B**). The peripheral C_60_ molecule marked by the arrow also shows a two-lobe motif in (**B**).

When a small amount of C_60_ molecules was deposited on the Cd(0001) surface at 100 K, they formed individual small clusters. Figure 1A shows a typical C_60_ heptamer (seven-molecule cluster) with one central C_60_ molecule surrounded by six peripheral molecules. At the bias of 1.2 V, except for the central molecule with a dim protrusion, the other six peripheral C_60_ molecules revealed a similar bright contrast. When the bias was reduced to 0.5 V, the upper-right C_60_ molecule marked by the arrow became dim and revealed a two-lobe shape (Figure 1B). We speculate that the main mechanism for the contrast changes of this molecule arises from the modification of the molecular orientation during low-bias scanning. To the best of our knowledge, such forms of isolated C_60_ heptamers were not reported in previous experiments.

### 2.2. Wavy Structure of the C_60_ Submonolayer Appears at 100 K

**Figure 2:**
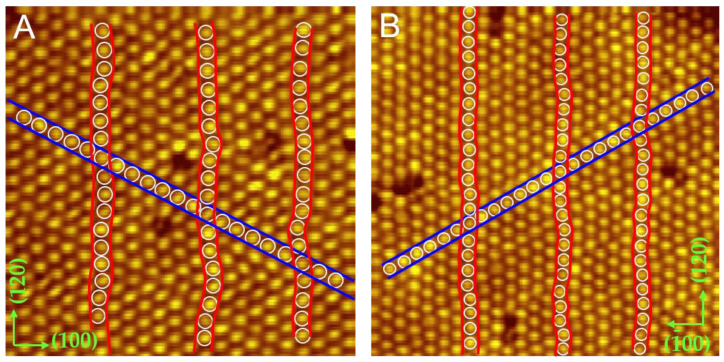
Wavy structure of the C_60_ submonolayer driven by compressive strain. (**A**) A wavy domain showing the wavy arrangement in the (120) direction, and the straight arrangement in the (1$\bar{1}$0) directions, 0.9 V, 20 × 20 nm. Both directions correspond to the √3 directions of the Cd(0001) surface. (**B**) Another domain showing the wavy alignment of C_60_ molecules, 0.4 V, 22.7 × 22.7 nm. The direction of straight alignment has an angle of 60 degrees with respect to that in (**A**).

As molecule coverage increased, an unexpected wavy structure of the C_60_ submonolayer appeared. Figure 2A is the STM image of a wavy domain where the C_60_ molecules in the (120) direction present a wavy arrangement while the molecules in the (1$\bar{1}$0) direction are arranged in a straight line. Both directions correspond to the √3 directions of the Cd(0001) surface. The three rows marked with red curves demonstrate the wavy arrangement clearly. Figure 2B displays another wavy domain and the direction of straight alignment has an angle of 60 degrees with respect to Figure 2A. The intermolecular spacing is 9.8 ± 0.1 Å in the straight rows and 9.4 ± 0.1 Å in the wavy rows. Both are apparently smaller than the preferred spacing (10.02 Å) of the (111) plane in fcc C_60_ crystals [48], indicating a 2% compressive strain in straight molecular rows, and a 6.3% compressive strain in wavy molecular rows.

When C_60_ submonolayers were subjected to external forces, C_60_ molecules correspond with stable orientations to attach to the substrate. However, it was not enough to release the stress by orientation and a wavy structure appeared. Driven by the compressive stress, the individual C_60_ molecules deviated from linear arrangement, and the molecular rows became wavy in order to release the strain. In early studies, when the molecule-substrate force worked as a dominant role, slightly curved stripes were also observed in C_60_ films deposited on an Si(111) 4 × 1 In surface [49]. In addition to unidirectional wavy structures, we observed another strain relaxation pattern through the formation of edge dislocations in the C_60_ monolayer.

### 2.3. High-Resolution Topological Graph of the Edge Dislocation

**Figure 3:**
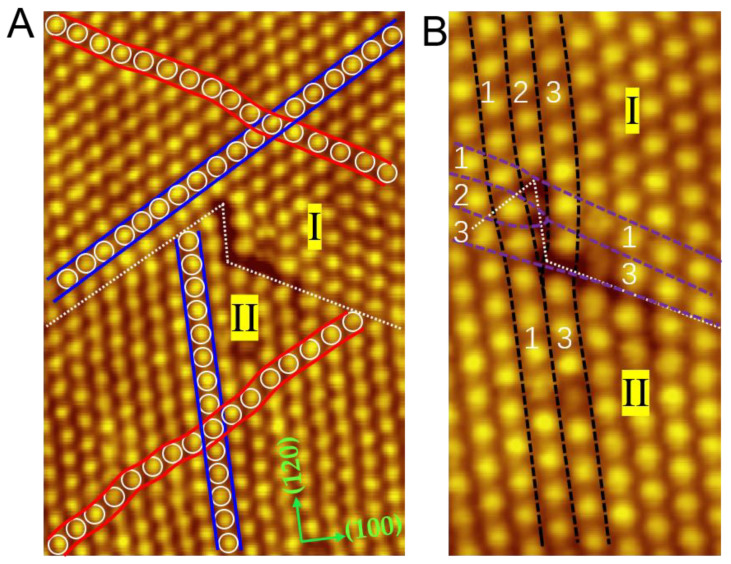
Two neighboring wavy domains (I and II) with different directions for the straight alignments. (**A**) Edge dislocation appears at the domain boundaries (white dotted lines), 23 × 15 nm, 2.8 V. (**B**) Close-up view of the edge dislocation as marked by the black and purple dotted curves, 7.5 × 13.5 nm, 3.0 V.

Figure 3A shows another area of the wavy structures comprising two neighboring wavy domains (I and II) with different directions for the straight alignments. The straight arrangements and wavy arrangements in Domain I and II are marked with blue curves and red lines, respectively. The straight arrangement in Domain II is in the (120) direction, while the straight arrangement in Domain I is in the (210) direction. When these domains encounter each other, an edge dislocation appears at the domain boundaries, tagged with white dotted lines. To the best of our knowledge, edge dislocation is seldomly precisely observed in molecular self-assembly. A similar phenomenon was achieved in the work of Klyachko, who found the edge dislocation in the third layer of C_60_ films grown on Ge(100) [31]. Unfortunately, the detail of the edge dislocation was hard to obtain due to the absence of a high-resolution STM diagram.

Figure 3B depicts the close-up view of the edge dislocation. Represented by black dotted lines, Domain I has an extra C_60_ row compared to Domain II. The same situation also occurs along the purple dotted lines. This observation indicates that the edge dislocation may occur in the monolayer regime and provides an intuitive STM diagram for the study of edge dislocations. Moreover, we noticed that this type of dislocation is absent when Cd(0001) substrate is kept at RT during C_60_ deposition.

### 2.4. Two Regular Domains in R26° and R33° 

**Figure 4:**
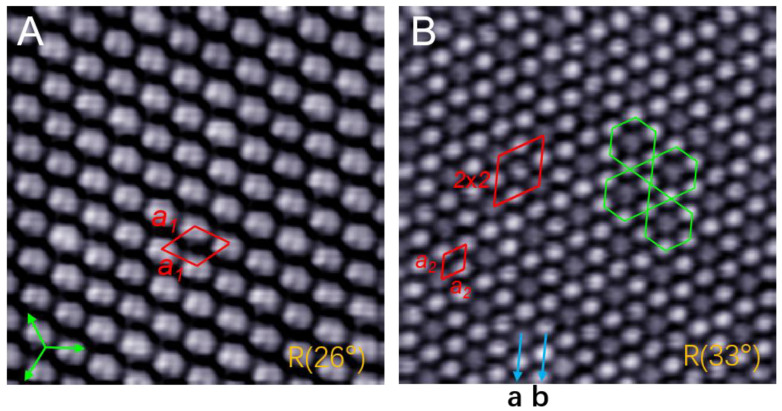
Two regular domains were obtained by deposition at room temperature. (**A**) Homogeneous orientation of C_60_ molecules in a R26° domain, 1.2 V, 10 × 10 nm. (**B**) The symmetric kagome lattice which is marked by hexagons in green reveals a 2 × 2 superstructure in an R33° domain, 2.0 V, 15 × 15 nm. Arrow **b** represents the molecular rows with bright molecules, and arrow **a** represents the rows with one bright and one dim molecules alternately arranged.

When growth temperature is raised to RT, two types of domain appear in the C_60_ monolayer. Figure 4A shows the topography of a R26° domain, in which all the C_60_ molecules adopt the same orientation. Each molecule reveals a two-lobe contrast corresponding to the C_60_ orientation with a 6:6 bond facing upward, similar to the C_60_ molecule in Au(111) and graphene [4,22]. The molecules in this domain exhibit a hexagonal lattice constant *a*_1_ = 10.0 ± 0.1 Å, close to the same value (10.02 Å) in C_60_ crystals [48]. This means that there is almost no strain when C_60_ molecules adopt the same orientation. Figure 4B shows a R33° domain that is composed of symmetric kagome lattices. Inside this domain, each dim C_60_ molecule is surrounded by six bright molecules, constituting a head-to-head arrangement of hexagonal rings. In other words, the C_60_ monolayer is composed of two types of molecular rows: In row-**b**, all the molecules show a bright protrusion, while in row-**a**, one bright and one dim C_60_ molecule alternately appear. As a result, the bright and dim molecules constitute a 2 × 2 superstructure, with each unit cell containing four molecules. The measured intermolecular distance is *a*_2_ = 10.2 ± 0.1 Å, indicating a 2% tensile strain in the R33° domain. These types of symmetric kagome lattices also exist in the reported C_60_ domains in Au(111) [8,23].

### 2.5. A larger 4 × 4 Superstructure of Kagome Lattice in an R44° Domain

**Figure 5:**
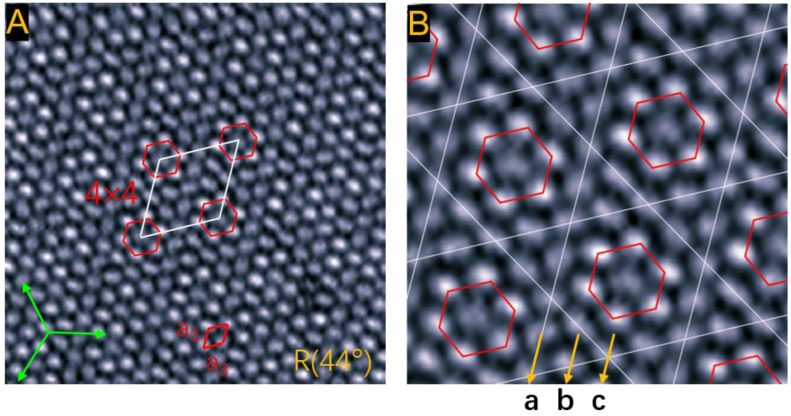
Kagome lattice formed at 310 K. (**A**) Symmetric kagome networks reveal a 4 × 4 superstructure in an R44° domain. The rhombus shows the primitive cell of the superstructure. 20 × 20 nm, 1.2 V. (**B**) Close-up view of the kagome network with each unit cell containing sixteen C_60_ molecules, 1.2 V, 10 × 10 nm. The molecular rows **a**, **b**, and **c** contain dim molecules, alternate two bright and two dim molecules, alternate one bright and one dim molecule, respectively.

When the growth temperature rises to 310 K, the C_60_ monolayer presents a larger kagome lattice with a 4 × 4 superstructure, shown in Figure 5A. The intermolecular distance is *a*_3_ = 10.5 ± 0.1 Å, much larger than the value (10.02 Å) in C_60_ crystals. The tensile strain is as large as 5%. These properties indicate that the packing density of C_60_ molecules decreases with an increase in growth temperature due to thermal expansion of the C_60_ layer. As displayed in Figure 5B, the six trigonal regions distribute symmetrically around the central hexagon, constituting the kagome lattice. Each kagome unit cell contains sixteen molecules: a C_60_ heptamer located at the inner hexagon and nine peripheral molecules. The heptamer consists of a central dim molecule and six bright surrounding molecules. We noticed that the C_60_ monolayer in this domain is composed of three types of molecular rows: all C_60_ molecules in row-**a** show a dim contrast; two dim molecules and two bright molecules are alternately arranged in row-**b**; one bright and one dim molecule appear alternately in row-**c**.

## 3. Materials and Methods

The experiments were performed in an ultra-high vacuum low-temperature scanning tunneling microscopy (Unisoku, Japan) with a base pressure close to 1.5 × 10^−10^ Torr. A clean surface of Si(111)-7 × 7 was obtained through thermal flashing to 1550 K after uninterrupted degassing at 820 K for more than 7 hours. The flat and smooth Cd(0001) thin film was treated by depositing 15-20 monolayers onto the surface of Si-7 × 7. C_60_ molecules were evaporated from a homemade Tantalum boat at a rate of 0.4 ML/min onto the Cd(0001) thin films. After the process was completed, C_60_/Cd/Si was transferred into the STM observation chamber. The entire data were obtained in a constant current mode at 78 K (liquid nitrogen temperature).

## 4. Conclusions

In summary, our research showed the morphology of C_60_ molecules on Cd(0001) substrate in three different growth temperatures (100 K, RT, and 310 K). STM studies demonstrated that C_60_ molecules are bright or dim in the thin film. At 100 K, we found the isolated C_60_ seven-molecule cluster, which indicates that the C_60_ heptamer can exist alone. As the molecule coverage increased, an unexpected wavy structure appeared, which suggests the C_60_ submonolayer was subjected to a large compressive strain. As a normal reaction, when under large external stress, the film of atoms or molecules may present larger bulk density or form superstructures. In this study, the arrangement of C_60_ curved instead of the aforementioned action. Additionally, an edge dislocation was captured when two different wavy structures encountered each other, which may develop a new phenomenon to study edge dislocation. Two types of symmetric kagome lattices were found at elevated growth temperatures, the 2 × 2 superstructure in the R33°domain with a 2% tensile strain at RT, and the 4 × 4 superstructure in the R44° domain with a 5% tensile strain at 310 K. Our results provide a new routine to fabricate and investigate pressure-resistant materials.

## Data Availability

The data presented in this study are available on request from the corresponding author.
